# Introductory

**Published:** 1879-12

**Authors:** 


// HALL'S JOURNAL OF HEALTH.

E. H. GIBBS, A. M., M. D., EDITOR.

DECEMBER, 1879.

THIS number completes the Twenty-sixth Year of HALL'S JOURNAL OF HEALTH. We are conscious of having neglected to do many things that might have been done for the interests of our readers and ourselves; but we will "let the dead past bury its dead," and resolve to do better in the future. In spite of adverse circumstances which have seriously affected nearly every material interest during the past six years, this Journal has had an era of prosperity it never before enjoyed to the same extent. Its circulation has steadily increased, and it probably numbers among its readers a larger percentage of educated and refined people than any other American publication, because it is such persons, principally, who realize the importance of preserving the health. There is no similar publication that is so well and so favorably known, and so largely quoted from.

Other health publications have been started from time to time, but they have dwindled and died, after a brief existence, leaving the field which we were the first to occupy, and which we expect to retain without serious competition; not because we would not welcome such publications, but because the masses of the people think very little about their health until they lose it; consequently it would require a large capital and years of persevering industry to establish another Health Journal on a paying basis.

We intend very soon to enlarge the Journal, so that after printing the usual number of Health articles, we shall have room for miscellaneous articles of general interest; for gems of thought, in prose or verse, and for an occasional story of the better class, mingled with a little fun to give it all spice and interest. It is indeed intended to make HALL'S JOURNAL OF HEALTH a publication for the family, that will always be looked for with interest, and that will take the place of publications of a less refining influence, that too often find their way into the household, for the want of something better. We believe that every copy of the "Journal" will be worth a year's subscription to any person who is thoughtful enough and prudent enough to follow its counsels. The subscription price is $1.50 a year. Agents can retain 50 cents out of each subscription, sending us $1.00. We make no exceptions to these terms in any case. Address HALL'S JOURNAL OF HEALTH, 141 Eighth street, New York.

